# A Narrative Review of Medicare Part D Medication Therapy Management Program Design Challenges and the Development of an Evidence-Informed MTM Benefit Program for a Large Self-Insured Employer

**DOI:** 10.3390/pharmacy14070149

**Published:** 2026-10-11

**Authors:** Lama Al Damen, Alan J. Zillich, Karen Suchanek Hudmon, Geoffrey M. Curran, Margie E. Snyder

**Affiliations:** 1 Department of Pharmacy Practice, College of Pharmacy, Purdue University, 575 Stadium Mall Drive, West Lafayette, IN 47907, USA; laldamen@purdue.edu (L.A.D.); azillich@purdue.edu (A.J.Z.); khudmon@purdue.edu (K.S.H.); 2 Central Arkansas Veterans Healthcare System, Little Rock, AR 72205, USA; currangeoffreym@uams.edu; 3 Department of Psychiatry, University of Arkansas for Medical Sciences, Little Rock, AR 72205, USA

**Keywords:** medication therapy management, Medicare Part D, community pharmacy, implementation science, implementation outcomes, comprehensive medication review, electronic health records, pharmacy practice

## Abstract

The Medicare Part D Medication Therapy Management (MTM) program has several well-documented challenges, including restrictive eligibility criteria, low patient engagement, and difficulties integrating MTM services into pharmacy workflows and broader healthcare teams. These challenges are often driven by inadequate health information technology infrastructure, limited coordination between pharmacists and prescribers, and inefficiencies in standardized documentation for Comprehensive Medication Reviews (CMRs). This narrative review synthesized published literature to identify challenges and limitations of the Medicare Part D MTM program and used Purdue University’s MTM program design documentation to compare program features and describe the evidence and rationale underlying key design decisions. Purdue’s MTM program incorporated broader patient eligibility, more frequent MTM services, and provider-developed communication templates to address limitations in patient access, program uptake, and workflow feasibility identified in the Medicare Part D program. However, challenges related to optimization of standardized CMR documentation and integration with electronic health records (EHRs) remained unresolved. Overall, this review demonstrates how evidence-informed program design may strengthen MTM implementation while identifying opportunities for future refinement. Future evaluation of the Purdue MTM program will assess its acceptability, feasibility, adoption, reach, and fidelity.

## 1. Introduction

Medication therapy management (MTM) is defined as “a distinct service or group of services that optimize therapeutic outcomes for individual patients” [1]. Specifically, MTM is conducted to evaluate patients for medication therapy problems (MTPs) by assessing their medications for appropriateness, safety, and effectiveness while ensuring patients can be adherent to their prescribed regimens. In 2006, the U.S. Centers for Medicare & Medicaid Services (CMS) launched outpatient prescription drug benefits under Medicare Part D; CMS disseminates guidance to Part D plans defining the required MTM services and MTM eligibility criteria for targeted beneficiaries, along with information regarding required documentation for the services [2].

Medicare is the federally administered health insurance program that primarily covers adults aged 65 years and older and certain younger individuals with disabilities in the United States. Medicare Part D is the component through which private insurance plans approved by the federal government provide outpatient prescription drug coverage [1]. These plans are required to offer MTM services to eligible beneficiaries who meet specified clinical, medication-use, and cost-related criteria [2]. 

Targeted beneficiaries for the Part D MTM program are enrollees who meet the characteristics of at least one of the following two groups: (a) individuals with multiple chronic diseases, taking multiple Part D drugs, and likely to incur annual costs for covered Part D drugs greater than or equal to the specified annual MTM cost threshold, or (b) beneficiaries with an active coverage limitation for being at-risk for prescription drug abuse or misuse. In 2025, CMS expanded Part D MTM eligibility for the former by updating the annual threshold cost for covered Part D medications and adding HIV/AIDS as a core chronic condition [2].

Medicare Part D plan sponsors must, at a minimum, offer an annual comprehensive medication review (CMR) and quarterly targeted medication reviews (TMRs) for all beneficiaries enrolled in the MTM program. The CMR is a comprehensive, real-time, interactive medication review and consultation with the beneficiary and/or their caregiver that assesses medication use for the presence of MTPs. The TMR is a focused review that targets specific MTPs or potential problems rather than reviewing the patient’s entire medication profile. MTM services for Part D must be delivered by a pharmacist or other qualified provider [2].

Medicare Part D also requires that MTM beneficiaries receive an individualized, written summary in a standardized format following each CMR. The written summary in the standardized format includes discussion of the beneficiary’s concerns with their drug therapy; the purpose and instructions for using their medications; review of a beneficiary’s medications, including prescription drugs, non-prescription drugs, and supplements, to aid in assessing medication therapy; and engaging beneficiaries in management of their drug therapy [3].

Following the delivery of MTM services, the MTM provider often offers recommendations to beneficiaries’ prescribers to resolve MTPs or identify opportunities to optimize the targeted beneficiaries’ medication use. These prescriber consultations may be passive (e.g., faxed or mailed) or interactive using face-to-face or telephonic modes of communication.

As an employer, Purdue University provides insurance coverage to approximately 24,000 employees under its benefits programs with medical, prescription, dental, and vision benefits. Its health insurance plans include wellness programs, preventive checkups, prescription drugs, clinic visits, hospital stays, and emergency department visits, with cost-sharing based on provider networks and plan choice. As part of a wellness program, access to MTM, which was already available via the on-campus Center for Healthy Living in West Lafayette, Indiana, was expanded in May 2024 and in place for approximately one year as a “pilot.” This expansion included contracting with an MTM vendor to deliver MTM services across Indiana through its participating network of community pharmacies. This benefit was available to all enrolled employees and covered dependents on a Purdue health plan with no cost to the patient for MTM services [4].

The Purdue MTM program differed from the Medicare Part D MTM program in several aspects of its design. These differences were intended to address challenges identified in the design and delivery of Medicare Part D MTM services and to align the Purdue program with evidence-informed MTM practices.

The objectives of this narrative review were to (a) describe challenges and limitations associated with the design and delivery of Medicare Part D MTM services, particularly in community pharmacy settings; (b) compare the designs of the Medicare Part D and Purdue University MTM programs; and (c) describe the evidence and rationale underlying the design decisions adopted in the Purdue MTM program. The review is organized into three sections. The first examines challenges associated with the design and delivery of Medicare Part D MTM services using evidence from Medicare Part D and other U.S.-based MTM programs. The second compares the design features of the Medicare Part D and Purdue MTM programs and explains the rationale underlying the Purdue program’s design decisions. The third discusses the limitations of the Purdue MTM program design.

## 2. Narrative Review Methods

A narrative literature review was conducted to identify and synthesize evidence concerning the design, delivery, implementation, and evaluation of medication therapy management (MTM) programs in the United States. PubMed and Google Scholar were searched for English-language publications published between 2009 and 2026. Search terms included combinations of “medication therapy management,” “Medicare Part D,” “comprehensive medication review,” “targeted medication review,” “community pharmacy,” “MTM eligibility,” “health disparities,” “patient engagement,” “pharmacist workflow,” “interprofessional collaboration,” “MTM documentation,” and “MTM implementation.” Reference lists of relevant publications were also reviewed to identify additional sources, including pertinent publications published before 2009. Government reports, policy documents, and program guidance issued by CMS were included when they provided information relevant to Medicare Part D MTM requirements, implementation, or evaluation.

Publications were considered for inclusion if they addressed the design, delivery, implementation, or evaluation of MTM programs in the United States. Eligible sources included studies of Medicare Part D MTM programs, as well as studies of MTM programs implemented in other U.S. settings when they provided evidence concerning implementation challenges. Topics of interest included eligibility and access, pharmacist remuneration, patient awareness and engagement, service uptake, pharmacy workflow, staffing and training, service frequency and follow-up, interprofessional collaboration, health information technology, documentation, health disparities, and clinical or economic outcomes. Publications were excluded if they did not examine MTM services in the United States or did not provide information relevant to the review objectives.

Publications were selected according to their relevance to the review objectives. Findings were organized into the principal MTM program-design domains identified across the literature. When available, findings were compared across empirical studies, government evaluations, policy documents, and program guidance. The resulting synthesis was used to describe the rationale underlying the Purdue MTM program’s design features.

Information about the Purdue University MTM program was obtained from program design documentation, vendor materials, communication templates, benefit descriptions, and discussions with individuals involved in the program’s development. These sources were used to describe the program’s features and the rationale underlying its design; they were not considered evidence of the program’s effectiveness.

A narrative review approach was selected because the evidence relevant to MTM program design and implementation was methodologically and conceptually diverse. The available literature included quantitative and qualitative studies, surveys, program evaluations, policy documents, government reports, and regulatory guidance addressing different populations, practice settings, implementation barriers, and clinical and organizational outcomes. Accordingly, the purpose of the review was not to estimate a pooled intervention effect or comprehensively describe the entire MTM literature, but to identify recurring design and implementation challenges and synthesize evidence relevant to the development of a context-specific MTM program. Because the review included heterogeneous source types and was not conducted as a systematic review or meta-analysis, no formal risk-of-bias assessment or evidence-grading framework was applied. This limits the strength of the conclusions that can be drawn from the synthesis.

### 2.1. Medicare Part D MTM Program Challenges

(1) Part D plans are not fully incentivized to offer a robust MTM program, resulting in problematic design decisions and inconsistent patient and healthcare utilization outcomes.

The current CMS bid structure for funding the Medicare Part D MTM program does not adequately support the development of comprehensive and effective MTM program designs. Plan sponsors are expected to deliver MTM services within their administrative budgets and do not receive payments specifically for these services [2,5]. As a result, Part D plan sponsors often lack clear and immediate financial benefits to invest in MTM programs. Financial incentives for the Medicare Part D MTM program’s performance are tied to higher Medicare Part D Star Ratings, primarily through the “CMR completion rate” measure. However, the Star Rating system does not directly reflect broader MTM quality or patient-centered outcomes [6]. Additionally, Medicare Part D programs only cover medication costs and do not cover expenses related to hospitalizations or clinical visits [2]. As a result, plan sponsors might not benefit from cost savings outside of the pharmacy (such as reduced hospital admissions or fewer physician visits) and tend to prioritize controlling drug spending rather than other activities that lead to longer-term clinical outcomes, which MTM could impact. Furthermore, the lack of compensation for pharmacists’ MTM services beyond mandatory ones and the inadequate financial incentives for the mandatory services represent additional challenges [7]. Thus, pharmacists might prioritize other compensated activities over providing MTM services due to inadequate financial incentives for providing these reviews.

In light of these insufficient and misaligned incentives, Part D plans have adopted a program design that has restrictive eligibility and less-than-optimal mandatory MTM services. The consequences were reflected in the CMS fact sheet of 2016, which reported that the program “fell short of its full potential to improve therapeutic outcomes and reduce unnecessary medical expenditure” [8]. At the same time, several studies revealed mixed therapeutic and healthcare utilization outcomes for Medicare Part D MTM beneficiaries. For therapeutic outcomes, one study showed that among older beneficiaries with Alzheimer’s disease, CMRs improved medication adherence for diabetes, hypertension, and hyperlipidemia [9]. Similarly, the final evaluation report for Medicare Part D MTM in chronically ill populations in 2013 showed that MTM was associated with enhanced medication adherence among patients with diabetes, chronic obstructive pulmonary disease, and heart failure. Furthermore, MTM programs demonstrated improved safety of drugs prescribed to new enrollees in the first 6 months. However, these positive effects lessened or reversed within one year after enrollment. For healthcare utilization, hospitalization was reduced for patients with diabetes or heart failure who received a CMR, but this was not observed among patients with chronic obstructive pulmonary disease [10]. Another study suggested that among patients with mental health disorders, reduced hospitalizations and emergency department visits were observed during the same year in which a CMR was received [11].

As a result, the CMS Center for Medicare and Medicaid Innovation launched the Part D MTM enhanced model in 2017 with a five-year testing plan. This model provided participating Medicare Part D sponsors with more flexibility in designing and targeting MTM programs beyond the CMS-defined criteria, provided a plan-specific prospective payment to support more extensive MTM interventions outside of a plan’s annual Part D bid, and provided incentives to programs showing cost-saving performance [8]. As indicated by the CMS evaluation reports published in 2019 [12] and 2021 [13], participating Part D sponsors broadened eligibility criteria, resulting in a significant increase in beneficiaries eligible for MTM services, but with few positive impacts on healthcare utilization outcomes. Additionally, despite the expanded reach, the model did not achieve statistically significant reductions in gross Medicare expenditures.

(2) Eligibility decisions have resulted in racial and ethnic disparities in MTM services eligibility, enrollment, and offering.

Over the years, a body of evidence has amassed describing potential racial and ethnic disparities in the Medicare Part D MTM program. Wang et al. [14,15] reported that based on the 2006, 2008, and 2010 minimum cost thresholds set by CMS, Black and Hispanic patients are less likely to be eligible for MTM services. However, Coe et al. reported that, based on actual 2013 and 2014 MTM program enrollment data, Black, Hispanic, and North American Native beneficiaries were more likely to be enrolled in MTM as compared to their White counterparts [16]. To understand the discrepancy between eligibility and enrollment, a review by Allen et al. [17] reported that enrollment significantly differed by race/ethnicity from the theoretical minimum thresholds stated by CMS. In addition to addressing racial disparities in enrollment, Coe et al. [16] evaluated disparities in CMR offering and receiving/completion. The study found that eligible Black and Hispanic older adults had lower odds of being offered a CMR when compared with White beneficiaries. Regarding CMR completion, Coe et al. [16] reported that the rate for CMR completion varied among beneficiaries who were enrolled in MTM and subsequently offered a CMR. Black older adults were more likely to receive a CMR compared to White older adults, whereas Asian, North American Native, and Hispanic older adults were less likely to receive it. Similarly, it has been shown that Asian, Hispanic, and North American Native patients (compared to White patients) had a lower likelihood of completing a CMR among the population deemed MTM-eligible by plans from 2013 to 2020 [18].

Besides evaluating racial disparities in Medicare Part D program eligibility, enrollment, CMR offering, and completion, another study [19] evaluated disparities in duration between MTM enrollment and CMR offering, opt-out rate, and the rate of continuation of CMR from the previous year. In this study, the time elapsed between MTM enrollment and CMR offering for Black, Hispanic, and Asian patients was longer than for White patients. Additionally, compared with White patients, the odds of opting out of MTM were higher for Black, Hispanic, and Asian patients, and the odds of continuing MTM from the previous year were lower for Black patients and higher for Asian patients (no difference for Hispanic patients). Based on these findings, it is apparent that racial and ethnic disparities exist and are a potential limitation in the Medicare Part D program.

(3) The frequency of CMRs and TMRs results in minimal opportunity for patient follow-up.

MTM services are intended to assess a patient’s medication to identify any potential MTPs and optimize patient therapy. Limiting required services to one annual CMR and four quarterly TMRs have inherent challenges for Medicare Part D MTM providers, as there is no mandatory and compensated follow-up for many MTPs identified during the mandatory sessions. Without follow-up, MTPs identified may remain unresolved, increasing the risk of poor outcomes such as hospitalizations, adverse events, or therapy failure. Pharmacist involvement in conducting medication reviews, with a 2-month follow-up, has been shown to improve outcomes with respect to decreased hospitalizations in the following year [20]. The MTM practice maturity level (i.e., early maturity versus later maturity, with better scores on the assessment of chronic illness care instrument, larger patient volume, more staff support, and more diverse payer mix) has also been identified, in qualitative research, as being associated with frequency of delivery of MTM services and follow-up [21].

(4) Difficulty integrating MTM services within the pharmacy workflow.

Many U.S.-based community pharmacists have expressed great difficulty in integrating MTM services within their existing workflow [22]. Pharmacists often struggle to determine whether MTM services should be integrated into their usual dispensing process or offered separately as a stand-alone consultative service. Additionally, the payers or MTM vendors require pharmacists to use a particular billing and/or documentation system that differs from those used by pharmacy dispensing systems [22]. Adding to these factors, community pharmacists are often overburdened with dispensing tasks [23], which could leave little time for patient-centered services like MTM. Furthermore, the lack of resources such as supportive pharmacy management, adequate staffing, and insufficient MTM training among pharmacists have been reported as barriers to providing MTM smoothly within the pharmacy workflow [7,22,24].

(5) Challenges with interprofessional collaboration during MTM delivery.

One of the key factors contributing to MTM’s success is communication between pharmacists and other healthcare providers. Research has demonstrated that comprehensive MTM services that coordinate drug therapy across healthcare providers can lead to improved patient outcomes [25]. This affords an enhanced opportunity for a comprehensive review of medications, identifying MTPs, ensuring effective recommendations are implemented, and aligning recommendations with the patient’s overall treatment plan. According to one study, community pharmacists viewed poor integration of medication management services within a coordinated healthcare team as the major barrier to improving patient health outcomes [24]. Another study highlighted “interprofessional relationship” barriers, which include poor access to patient medical information such as electronic health records (EHRs), poor access to primary care providers, lack of trusted professional relationships with providers, and lack of recognition of providers as important factors contributing to challenges in delivering MTM [26].

(6) Less-than-optimal standardized format for CMR documentation.

The standardized format for CMRs is intended to ensure consistency in the CMR by providing a template of expected information provided to patients. In one study, the perspectives of Medicare beneficiaries regarding the utility of a standardized format were evaluated. Fewer than half of the beneficiaries rated the standardized format as “very good” or “excellent” with respect to four key elements of medication management: (1) solving medication-related problems, (2) keeping track of medications, (3) correctly using medications, and (4) understanding why medications are being taken [27]. Furthermore, participants in the community-based, later-maturity-level practices reported dissatisfaction with CMS’ standardized format requirements, describing them as overwhelming for patients [21]. Additionally, qualitative research [28] suggests that beneficiaries and case managers preferred a “consolidated” standard format document that could be shared to update the broader healthcare team. Suggestions for improvement to the form included additional data fields capturing dosage, timing, drug interactions, cost, and less expensive alternatives [28].

(7) Lack of patient awareness and perceived low value for MTM services.

MTM’s success often relies on patient engagement, as many patients who are offered the service may not receive it or complete it. This could be attributed to patients’ lack of awareness of the service or its added value, and/or patients’ hesitancy to engage in these services. Patient awareness and perception of MTM services have been evaluated in different studies. In an internet-based survey conducted in 2007, 93% [29] of Medicare enrollees did not know about MTM. Most respondents believed that they did not need MTM services, and their expectation of the pharmacist’s role in providing MTM revolved around the drug product. Accordingly, most showed a very low willingness to pay for MTM services. Similarly, another survey conducted in 2006 with four regional community chain pharmacies in Maryland and Delaware indicated that 60% of respondents had never heard of MTM services, and 80% never had or received a medication therapy review [30]. These studies highlight patients’ lack of knowledge of the core elements of MTM services in community pharmacy settings. As a result, patients’ participation in these services may be limited.

### 2.2. Design and Rationale of the Purdue University MTM Program Compared with the Medicare Part D MTM Program

(1) Better incentivization for MTM service provision to increase MTM service uptake.

Purdue University adopted a service model that offered pharmacists a higher TMR revenue opportunity, compared to Medicare Part D. Because TMR could be performed at every medication fill, substantial opportunities for pharmacists to earn additional income while providing meaningful continuous monitoring of patient medications became possible. Given the volume of medication fills that occur in a community pharmacy setting, this model could lead to considerable financial reimbursements for pharmacists actively engaged in providing regular TMRs. Purdue also elected to pay for more frequent CMRs throughout the year.

Because insufficient financial compensation was among the most significant barriers to expanding MTM services in an outpatient setting [7], the enhanced reimbursement structure was intended to reduce financial barriers to pharmacists’ participation and enhance uptake of the services in community pharmacies. Whether this structure increased pharmacist participation or patient uptake within the Purdue program remains to be evaluated.

(2) Expansion of patient eligibility to reduce racial and ethnic disparities and to enhance beneficiaries’ awareness about MTM services.

In contrast to the eligibility requirements set for MTM services by Medicare Part D, the Purdue MTM program imposed no restrictions on enrolling beneficiaries in MTM services. Eligibility for TMRs included every patient filling one or more prescriptions at a community pharmacy that is part of the vendor’s network. For CMRs, all health plan members were eligible, with no mandatory selection criteria. However, individuals with specific characteristics (e.g., chronic conditions, higher drug expenditure) received targeted outreach from the on-campus wellness center to encourage participation.

Removing eligibility restrictions could help address the racial and ethnic disparities previously observed in the Medicare Part D MTM program [14,15,16]. By broadening eligibility for both TMRs and CMRs, a more diverse patient population can be reached, which could reduce racial and ethnic disparities in MTM access and outcomes.

Expanding eligibility, together with program outreach, was hypothesized to increase opportunities for patients to learn about MTM services and pharmacists’ roles as MTM providers. In one large national study, patients who were introduced to MTM expressed willingness to accept most components of the MTM service [31]. However, changes in awareness, perceptions, or participation have not yet been measured in the Purdue population.

(3) More frequent CMR and TMR to enhance patient follow-up and medication safety and efficacy.

The Purdue MTM program provides the same core MTM services as the Medicare Part D MTM plan. However, the Purdue program was designed to offer more frequent MTM services by encouraging TMR interactions to occur every time a prescription was dispensed at the affiliated network pharmacies. This decision was made to leverage the existing relationships between patients and their community pharmacist and to encourage continuous medication monitoring (CoMM) for MTPs. Prior literature describing the CoMM model found that when monitoring was provided with each fill, a high level of medication safety and effectiveness was maintained through identification of MTPs during the filling process [32]. Furthermore, after 12 months of implementing CoMM in a community pharmacy, claims data indicated that the average monthly costs per person among patients filling prescriptions at the CoMM pharmacy were $298 lower compared to patients who did not receive prescriptions at the CoMM pharmacy [33]. Moreover, encouraging TMRs to be conducted every time a prescription is filled could facilitate the integration of broader MTM services into the pharmacy workflow.

In the Purdue MTM program, patients did not receive TMRs if they filled prescriptions at a non-affiliated network pharmacy, but they still could receive CMRs. If using a network pharmacy, their community pharmacist provided the CMRs. Otherwise, pharmacists from the on-campus wellness center provided the CMRs telephonically. Specifically, up to five CMRs could be completed during a rolling 12-month period. The allowance of additional CMRs was intended to create opportunities for follow-up on MTPs identified during earlier encounters and for the detection of newly arising MTPs. Whether the additional encounters resulted in more timely resolution of MTPs or improved medication-related outcomes has not yet been determined.

(4) Enhanced communication with prescribers and Purdue members to provide more coordinated patient care.

Prior research suggests that pharmacist–physician collaboration may support medication management and care coordination [34]. In the Purdue MTM program, the foundations for the establishment of collaborative professional relationships with prescribers were elucidated in several ways. First, prescribers were consulted to help create standard communication templates that pharmacists were instructed to use to summarize their findings and recommendations for prescribers. Additionally, pharmacist–prescriber roles were coordinated by sending prescribers notification letters at the launch of the MTM program. These letters described expectations for MTM services and for prescribers’ responses to pharmacists’ recommendations. Also, health plan members were informed of the expanded MTM opportunity through announcements included in Purdue’s emailed newsletter as well as information added to the website. Table 1 compares key features of the Purdue University MTM program with Medicare Part D MTM program requirements and summarizes the rationale underlying Purdue’s design decisions.

### 2.3. Limitations of Purdue’s MTM Program Design

While the Purdue MTM program was designed to address many challenges identified with the Medicare Part D MTM program, some challenges remained unaddressed. First, the program did not meaningfully modify the content of the report provided to patients after a CMR to fully address all patient feedback on the standardized format, such as less expensive and alternative medications. Second, the Purdue MTM program did not address the challenges of technology integration. These challenges include the lack of integration of EHRs with pharmacy systems and the lack of integration of the pharmacy dispensing system with the MTM services documentation software. These challenges could create challenges for incorporating MTM services within the pharmacy workflow and within the healthcare team, particularly the comprehensive exchange of patient medical information with the prescribers. Third, the Purdue MTM program was developed within a single, large, self-insured university employer in Indiana. Its covered population, benefit structure, organizational resources, on-campus wellness services, participating pharmacy network, and relationships with local healthcare providers may differ from those of Medicare Part D plans, smaller employers, Medicaid programs, or other healthcare organizations. Its applicability outside the United States may be further limited by differences in healthcare financing, medication coverage, pharmacist remuneration, professional scopes of practice, regulatory requirements, and health information infrastructure. Therefore, the program design and its underlying rationale may not be directly generalizable to other populations, geographic regions, insurance structures, organizational settings, or national healthcare systems. Adaptation to local populations, resources, reimbursement structures, professional roles, regulations, and healthcare delivery environments may be necessary before implementing similar MTM program features in other settings.

## 3. Discussion

The present review describes the Purdue MTM program’s evidence-informed design and rationale for design decisions. Its design included enhanced financial incentives for pharmacists, broader eligibility, opportunities for more frequent MTM encounters, and prescriber-informed communication processes. These features were intended to address barriers reported in the Medicare Part D literature, including restrictive eligibility, limited patient follow-up, insufficient financial incentives, low awareness, and fragmented pharmacist–prescriber communication. However, it is not yet established whether these features improved service uptake, equity, clinical outcomes, or care coordination.

Nationwide, other MTM programs have been launched with differences in design from the Medicare Part D MTM program, and these programs yielded positive clinical and economic outcomes. For example, the Minnesota Medicaid MTM program (2013 criteria) provides services for patients who are taking prescription medication(s) to treat or prevent one or more chronic conditions. At the same time, the Minnesota program adopts specific criteria for follow-up encounters—e.g., for recipients diagnosed with a new (since the prior MTM session) chronic condition requiring treatment with two or more new medications [35].

However, the Purdue MTM program differed from Minnesota in eligibility and service design. Unlike the follow-up encounters in the Minnesota MTM program, the Purdue program adopted an open eligibility model, allowing any patient receiving a prescription medication to qualify for initial and/or follow-up MTM encounters. A further distinguishing feature of the Purdue model was aspects of outreach to physicians to promote a pharmacist–prescriber collaboration framework [35].

The Purdue experience highlights several program-design considerations that might be useful to employers, health plans, MTM vendors, and pharmacy organizations that are developing or refining MTM services. These organizations are encouraged to consider broadening eligibility beyond medication-cost and disease-count thresholds, permitting additional encounters when clinically appropriate follow-up is needed, aligning pharmacist reimbursement with the frequency and complexity of the services provided, and establishing standardized procedures for communicating recommendations to prescribers. However, the appropriateness and feasibility of these features will depend on the population served, benefit structure, pharmacy network, workforce capacity, reimbursement model, and available health information technology. Organizations adopting similar approaches should prospectively evaluate reach, adoption, feasibility, fidelity, service completion, resolution of medication therapy problems, healthcare utilization, cost, and equity to determine whether the selected design features achieve their intended effects.

Although Medicare Part D and employer-sponsored health insurance are specific to the United States, many of the implementation challenges discussed in this review extend beyond the U.S. healthcare system. Medication management programs across different settings must address fundamental questions concerning patient eligibility, provider remuneration, continuity of follow-up, integration into established workflows, and communication among pharmacists, patients, and other healthcare professionals. They must also address limitations in documentation and health information exchange. Accordingly, the Purdue program provides a context-specific example of how evidence concerning common implementation challenges can be translated into program-design decisions. Although its individual features may not be directly transferable to every healthcare system, the process of identifying barriers, establishing design priorities, and linking those priorities to specific program responses may be useful to organizations developing or adapting medication management services internationally.

## 4. Conclusions and Future Directions

The Purdue University MTM program integrated design decisions regarding the payment model, service eligibility and frequency, and communications with prescribers that differed from those that are characteristic of the Medicare Part D MTM program. However, some challenges remained unaddressed, including patient report documentation optimization and technology integration. Furthermore, research is needed to characterize the program’s preliminary outcomes.

Because the Purdue MTM program incorporated novel design components, the next step will be to evaluate implementation outcomes from the program’s pilot year. This includes assessing adoption, feasibility, acceptability, reach, and fidelity. These results could inform future iterations of an MTM program for Purdue as well as support other MTM program efforts.

## Figures and Tables

**Table 1:** Comparison of Medicare Part D Medication Therapy Management program requirements and the Purdue University MTM program design and rationale.

Program Feature	Medicare Part D MTM Program	Purdue University MTM Program	Rationale for Purdue Design
Covered population	Eligible Medicare Part D beneficiaries	Employees and covered dependents enrolled in a Purdue health plan	Extends MTM access to a self-insured employer population
Eligibility	Defined using chronic diseases, number of Part D drugs, projected annual drug costs, or at-risk beneficiary status	All covered health plan members eligible for CMRs; members filling at least one prescription at a participating network pharmacy eligible for TMRs	Reduce eligibility-related barriers and broaden access
CMR frequency	At least one CMR annually	Up to five CMRs within a rolling 12-month period	Permit additional assessment and follow-up when needed
TMR frequency	At least quarterly	Could occur with each prescription fill at participating network pharmacies	Support more continuous medication monitoring
Service delivery	Pharmacist or other qualified MTM provider; delivery models vary by plan	Participating community pharmacists or pharmacy at the on-campus wellness center	Use existing pharmacist–patient relationships while maintaining access for members not using a network pharmacy
Pharmacist reimbursement	Funded through the Part D plan’s administrative budget; reimbursement arrangements vary	Separate payment opportunities for CMRs and more frequent TMRs	Strengthen financial support for pharmacist participation
Prescriber communication	Approach varies across plans and providers	Standard communication templates developed with prescriber input and notification letters describing program expectations	Support consistent communication and care coordination
Technology integration	Integration with dispensing systems and EHRs varies and is often limited	No direct integration of MTM documentation, dispensing systems, and EHRs	Recognized as an unresolved implementation limitation
Evaluation status	Outcomes have been examined in multiple studies and CMS evaluations	Implementation and patient-level outcomes have not yet been formally evaluated	Future evaluation will assess implementation and outcome measures

## Data Availability

No new data were created or analyzed in this study. Data sharing is not applicable to this article.
